# Erratum to: Gauge-independent emission spectra and quantum correlations in the ultrastrong coupling regime of open system cavity-QED

**DOI:** 10.1515/nanoph-2022-0795

**Published:** 2023-01-05

**Authors:** Will Salmon, Chris Gustin, Alessio Settineri, Omar Di Stefano, David Zueco, Salvatore Savasta, Franco Nori, Stephen Hughes

**Affiliations:** Department of Physics, Engineering Physics and Astronomy, Queen’s University, Kingston, ON K7L 3N6, Canada; Department of Applied Physics, Stanford University, Stanford, CA 94305, USA; Dipartimento di Scienze Matematiche e Informatiche, Scienze Fisiche e Scienze della Terra, Università di Messina, I-98166 Messina, Italy; Instituto de Ciencia de Materiales de Aragón and Departamento de Física de la Materia Condensada, CSIC-Universidad de Zaragoza, Pedro Cerbuna 12, 50009 Zaragoza, Spain; Fundación ARAID, Campus Río Ebro, 50018 Zaragoza, Spain; Theoretical Quantum Physics Laboratory, RIKEN Cluster for Pioneering Research, Wako-shi, Saitama 351-0198, Japan; Physics Department, The University of MI, Ann Arbor, MI 48109-1040, USA

After the publication of our paper [1], the authors found that Figure 5(c) was using an incorrect data file, which has been corrected here.

**Figure 5:**
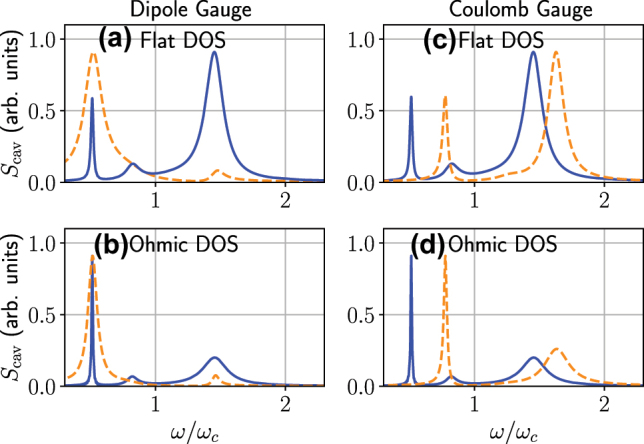
The computed cavity spectra using the dipole gauge (left) and Coulomb gauge (right), with a flat DOS [*κ* (*ω*) = *κ*, panels (a, c)] and an Ohmic DOS [*κ* (*ω*) = *κω*/*ω*_*c*_, panels (b, d)] using the generalized master equation [see Eqs. (A9)–(A10) in Appendix A]. In both cases, the effect of the gauge correction (solid lines versus dashed lines) is dramatic. We use the same parameters as in Figure 2 of the main text, with incoherent driving, and parameters *η* = 0.5 and *κ* = 0.25 *g*. Notably, in all cases, regardless of the spectral function, the corrected dipole gauge and corrected Coulomb gauge results are identical.
